# Metal-Organic Frameworks of MIL-100(Fe, Cr) and MIL-101(Cr) for Aromatic Amines Adsorption from Aqueous Solutions

**DOI:** 10.3390/molecules24203718

**Published:** 2019-10-16

**Authors:** Mao-Long Chen, Shu-Yang Zhou, Zhou Xu, Li Ding, Yun-Hui Cheng

**Affiliations:** College of Chemistry and Food Engineering, Changsha University of Science & Technology, Changsha 410114, China

**Keywords:** metal-organic framework, adsorption, aromatic amines, aniline, 1-naphthalamine, o-toluidine, 2-amino-4-nitrotoluene, 2-nitroaniline

## Abstract

MIL-100(Fe, Cr) and MIL-101(Cr) were synthesized by the hydrothermal method and applied to the adsorptions of five aromatic amines from aqueous solutions. These three metal-organic frameworks (MOFs) were well characterized by powder X-ray diffraction (PXRD), scanning electron microscope (SEM), transmission electron microscope (TEM), thermogravimetric analysis (TGA) and surface area analysis. The adsorption mechanism of three MOFs and the effects of the structures of MOFs on the adsorption of aromatic amines were discussed. The results show that the cavity system and suitable hydrogen bond acceptor were important factors for the adsorption for five aromatic amines of aniline, 1-naphthalamine, o-toluidine, 2-amino-4-nitrotoluene and 2-nitroaniline: (a) the saturated adsorption capacity of aniline, 1-naphthylamine and o-toluidine on MIL-100(Fe) were 52.0, 53.4 and 49.6 mg/g, respectively, which can be attributed to the intermolecular hydrogen bond interaction and cavity system diffusion. (b) The adsorption capacity of 2-nitroaniline and 2-amino-4-nitrotoluene on MIL-101(Cr) were 54.3 and 25.0 mg/g, respectively, which can be attributed to the more suitable pore size of MIL-101(Cr) than that of MIL-100(Fe, Cr). The MOFs of MIL-100(Fe) and MIL-101(Cr) can be potential materials for removing aromatic amines from aqueous solutions.

## 1. Introduction

As a typical organic compound, aromatic amines are widely used in the production of dyes, pharmaceuticals or resins [1]. In the industrial production of dyes, aromatic amines can flow into the water in large amounts, causing water pollution. Currently, azo dyes are widely found in textiles, paints, plastics and rubber. These dyes can be gradually degraded to produce aromatic amines (such as aniline), which are generally carcinogenic [2,3,4,5]. Even more frightening is that some azo dyes are also used for illegal food additives. For example, Sudan Red is illegally used in the food industry to increase the freshness of food [6], and Sudan red will decompose in the human body to produce aromatic amines, causing human body damage. Aromatic amines can also be formed in excessively cooking of protein-rich foods [7]. In addition, aromatic amines can be found in tobacco smoke at high concentrations [8,9,10]. Thus, it is necessary to remove aromatic amines efficiently.

MOFs represents a series of unique crystalline materials [11]. Metal ions and organic ligands together can form porous polymers through coordination bonds [12,13,14]. In terms of separation, the most important characteristics of MOFs are their large scale of controllable pore size and their selectivity to the size and shape of guest molecules [15]. Due to many types of ligands that can be used for constructing MOFs, the pore size of MOF can range from a few angstrom to a few nanometers [16,17]. Another advantage of MOFs is that they can introduce various functional sites within the frameworks or channels to highly regulate the interactions between subjects and objects. Therefore, these materials have great potential applications in adsorption and separation [18,19,20,21,22]. 

MILs is a subset of MOF and has received increasing attention due to its water and thermal stability [23]. MIL-100(Fe, Cr) and MIL-101(Cr) have similar zeotype cubic structures consisting of two types of cages and windows (pentagonal and hexagonal) whose dimensions are around 5 and 8 Å for MIL-100 and 12 and 16 Å for MIL-101(Cr), respectively [24,25,26,27,28,29]. As reported previously, the Fe(Cr) Lewis acid sites could be produced by the removal of the two coordinated water molecules of Fe(Cr) octahedra and the partial departure of anions through vacuum activation. Thus MIL-100(Fe,Cr) and MIL-101(Cr) can be expected to have a large amount of Fe(Cr) Lewis acid sites and have significant advantage for adsorption of alkaline chemicals such as aromatic amines [30,31,32,33]. However, in aquous solution, the Fe(Cr) Lewis acid sites may coordinate with water molecules again. Thus, for some harmful molecules in aquous solutions such as aromatic amines, suitable hydrogen bond acceptor and appropriate pore systems are identified as the key factors for adsorption. 

**Scheme 1:**
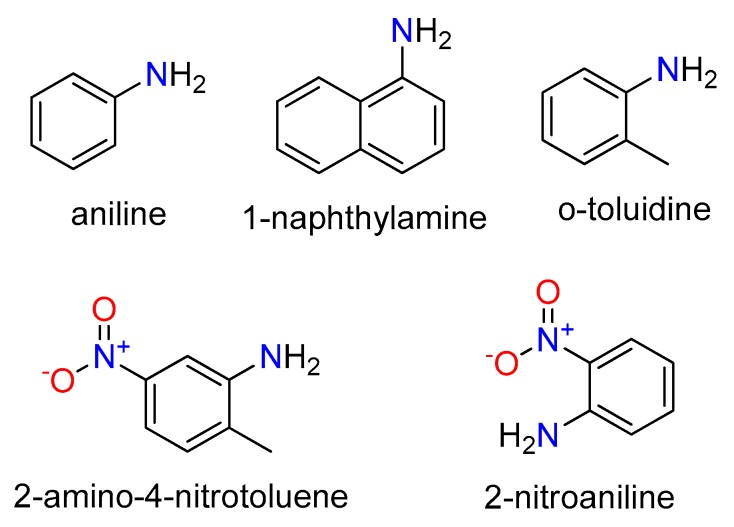
The molecular structures of aniline, 2-nitroaniline, o-toluidine, 1-naphthylamine and 2-amino-4-nitrotoluene.

In this paper, the adsorption of five aromatic amines were carried out in three typical MOFs. The molecular structures of these aromatic amines are shown in Scheme 1. The different adsorption results of these five aromatic amines on the synthesized MOFs were analyzed, indicating that MIL-100(Fe) and MIL-101(Cr) can be used as potential materials to remove aromatic amines from aqueous solutions.

## 2. Results and Discussion

### 2.1. Synthesis and Characterization

**Figure 1:**
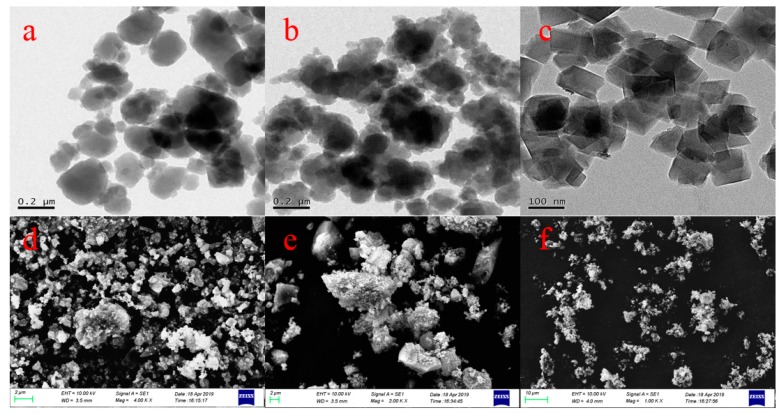
Transmission electron microscopy (TEM) images of (**a**) MIL-100(Fe) (**b**) MIL-100(Cr) (**c**) MIL-101(Cr) and Scanning electron microscopy (SEM) images of (**d**) MIL-100(Fe) (**e**) MIL-100(Cr) (**f**) MIL-101(Cr).

The particle sizes of the synthesized three materials are on the order of nano-sized as shown in the TEM image (Figure 1a–c). Particle sizes of MIL-100 (Fe, Cr) are around 200–300 nm, and the particle size of MIL-101(Cr) is about 100 nm. It can be clearly seen from the SEM image (Figure 1d–f) that all of the three MOFs exhibited microcrystals. FT-IR spectra (Figure S1) of MIL-100(Fe, Cr), MIL-101(Cr) are consistent with the reported spectras in these reports [32,34,35], which indicates the successful synthesis of MOF materials.

**Figure 2:**
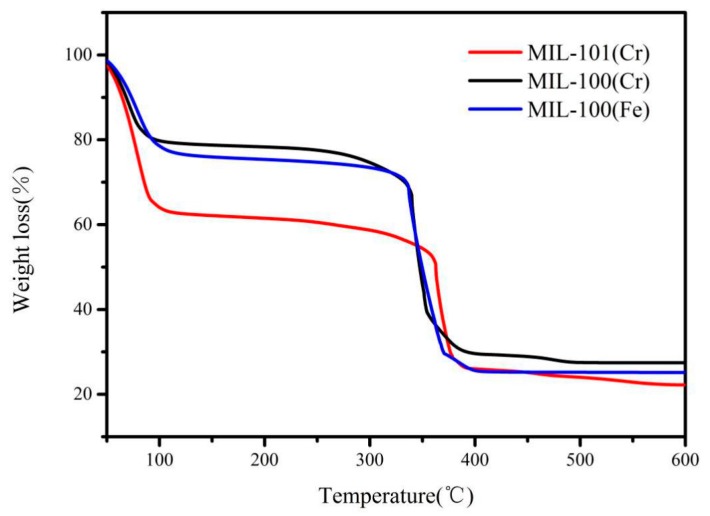
Thermal gravimetric analysis (TGA) profiles of MIL-100(Fe, Cr), MIL-101(Cr).

The thermogravimetric analysis curves for MIL-100(Fe), MIL-100(Cr) and MIL-101(Cr) (Figure 2) show two-step weight losses at 30 to 120 °C and 300 to 400 °C, respectively. The first loss can be attributed to the volatilization of guest molecules in the MOF structures. The second loss can be attributed to the pyrolysis of the ligands of the MOFs, causing the collapse of frameworks. These results are identical to the previously reported thermogravimetric analysis [30,36,37], indicating that MIL-100(Fe), MIL-100(Cr) and MIL-101(Cr) have high thermal stability. The TG-DTG curves and the detailed thermal analysis data are shown in Figure S2 and Table S1, respectively.

**Figure 3:**
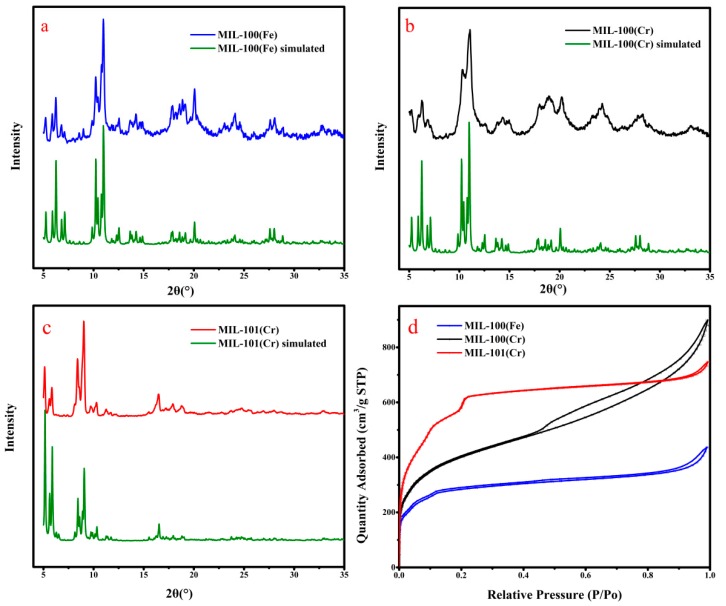
(**a**) XRD patterns of MIL-100(Fe); (**b**) XRD patterns of MIL-100(Cr); (**c**) XRD patterns of MIL-101(Cr); (**d**) N_2_ adsorption-desorption isotherms (at 87 K) of MIL-100(Fe) (blue), MIL-100(Cr) (black) and MIL-101(Cr) (red).

The PXRD patterns of MIL-100(Fe), MIL-100(Cr) and MIL-101(Cr) are consistent with the simulated data, indicating successful synthesis of MOFs too (Figure 3a–c)). Sharp diffraction peaks represent their high degree of crystallinity. The diffraction peaks of the three samples were sharp and intense, indicating their highly crystalline nature. BET analysis was conducted to examine the porous nature of the three MOF samples. N_2_ adsorption/desorption isotherms and pore-size distribution of three materials were shown in Figure 3d and Figure S3. Results of pore sizes indicated that they are all microporous materials (Table S2). MIL-101(Cr) has the largest BET surface area (2134 m^2^/g) and the largest pores (1.61 nm) in three MOFs. The BET surface area and pore size are 1018 m^2^/g and 1.14 nm for MIL-100(Fe), and 1427 m^2^/g and 1.26 nm for MIL-100(Cr), respectively. The MIL-100(Cr) show high surface area than that of MIL-100(Fe) which may be attributed to crystal size of MIL-100(Cr) was smaller than that of MIL-100(Fe), which can also can be find SEM image and PXRD patterns.

### 2.2. Adsorption Results

**Figure 4:**
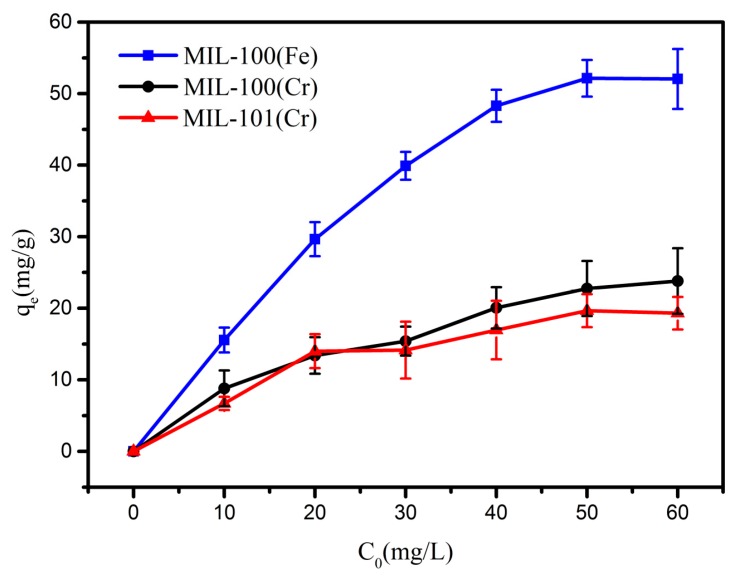
Adsorption isotherms for aniline adsorption on MIL-100(Fe) (blue), MIL-100(Cr) (black) and MIL-101(Cr) (red).

The calibration curves (Figure S4) were obtained from the standard solution in the range of 0–80 mg/g. The absorbance of aromatic amines gradually increased with the increasing of concentration which shown a R^2^ higher than 0.999. The adsorption results for each experiment are shown in Table S3. Figure 4 showed the isothermal adsorption curves of aniline on MIL-100(Fe, Cr) and MIL-101(Cr). It is apparent that all three MOFs of MIL-100(Fe, Cr) and MIL-101(Cr) show some adsorption to aniline. This phenomenon can be attributed to the fact that all three MOFs contain suitable hydrogen bond acceptor and pore systems for aniline [38]. However, MIL-100(Fe) has the largest adsorption capacity for aniline (52 mg/g).

**Figure 5:**
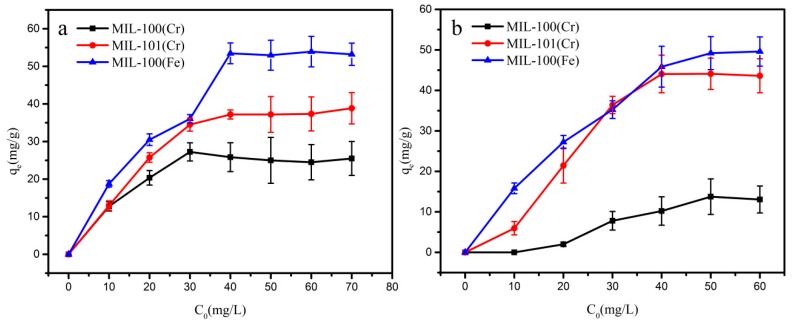
Adsorption isotherms for (**a**) 1-naphthylamine and (**b**) o-toluidine adsorption on MIL-100(Fe) (blue), MIL-100(Cr) (black) and MIL-101(Cr) (red).

Figure 5a shows the isothermal adsorption curves of 1-naphthylamine on MIL-101(Cr) and MIL-100(Fe, Cr). Similar to the case of aniline, MIL-100(Fe, Cr) and MIL-101(Cr) show some adsorption to 1-naphthylamine. MIL-100 (Fe) has the largest adsorption capacity of 1-naphthylamine to 53.4 mg/g. This phenomenon can be attributed to the formation of hydrogen bonds between MIL-100 (Fe) and 1-naphthylamine. Besides, it was noticed that MIL-101(Cr) adsorb a little more 1-naphthylamine than that of MIL-100(Cr) which can be ascribed to the more suitable pore size of MIL-101(Cr) than that of MIL-100(Cr). Adsorption of o-toluidine is similar to 1-naphthylamine (Figure 5b).

**Figure 6:**
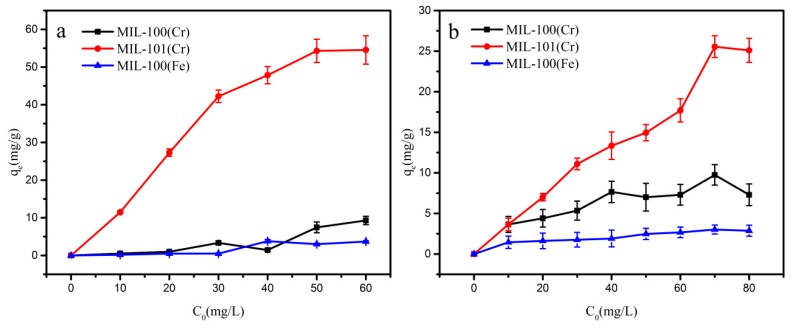
Adsorption isotherms for (**a**) 2-nitroaniline and (**b**) 2-amino-4-nitrotoluene adsorption on MIL-100(Fe) (blue), MIL-100(Cr) (black) and MIL-101(Cr) (red).

Figure 6a shows the isothermal adsorption curves of 2-nitroaniline on MIL-100(Fe,Cr) and MIL-101(Cr). Unlike aniline, MIL-100(Fe, Cr) show little adsorption to 2-nitroaniline. MIL-101(Cr) has the largest adsorption capacity of 2-nitroaniline to 54.3 mg/g. This phenomenon can be attributed to the more suitable pore size of MIL-101(Cr) than that of MIL-100(Fe, Cr). Adsorption of 2-amino-4-nitrotoluene is similar to 2-nitroaniline (Figure 6b).

### 2.3. Adsorption Performance

**Figure 7:**
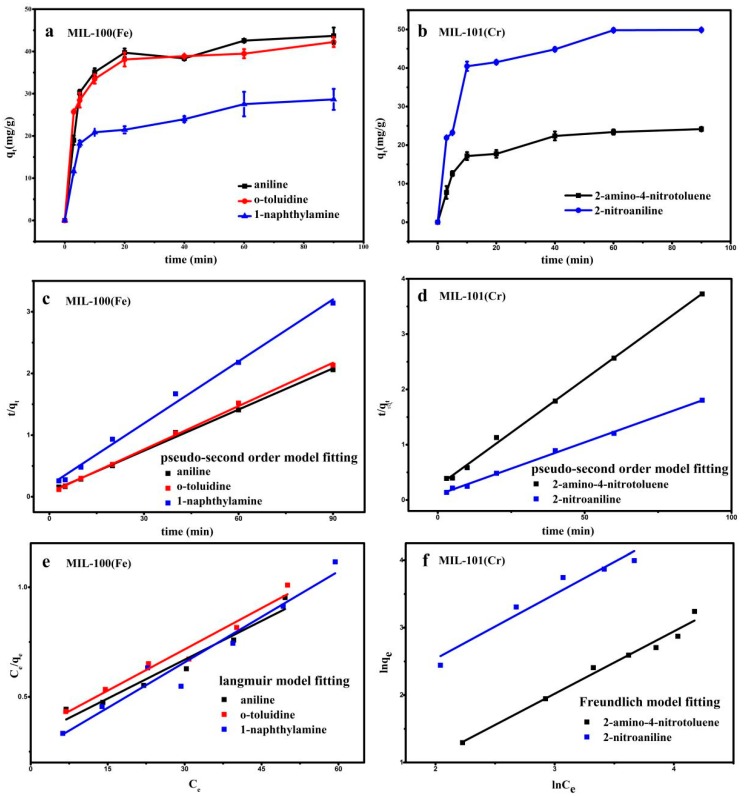
(**a**) The relationship between the amount of adsorption of MIL-100(Fe) and time. (**b**) The relationship between the adsorption amount of MIL-101(Cr) and time. (**c**) The pseudo second-order kinetics of MIL-100(Fe). (**d**) The pseudo second-order kinetics of MIL-101(Cr). (**e**) Isothermal Langmuir model of MIL-100(Fe). (**f**) Isothermal Freundlich model of MIL-101(Cr).

From the upper adsorption results, MIL-100(Fe) shown the highest adsorption for aniline, 1-naphthylamine and o-toluidine, and MIL-101(Cr) shown highest adsorption for 2-nitroaniline and 2-amino-4-nitrotoluene, thus only adsorption performance of MIL-100(Fe) and MIL-101(Cr) will be discussed in this section. The relationship between the adsorption amount and the contact time of the aromatic amine solution is shown in Figure 7a,b and the adsorption kinetic data is fitted by pseudo first-order kinetic model and pseudo second-order kinetic model. Its mathematical equation is as follows:

(1) $$\mathrm{Pseudo}\;\mathrm{first}-\mathrm{order}\;\mathrm{kinetic}\;\mathrm{model}:\;{\log(q}_{e}{-q}_{t})={\mathrm{logq}}_{e}-\frac{k_{1}}{2.303}t$$

(2) $$\mathrm{Pseudo}\;\sec\mathrm{ond}-\mathrm{order}\;\mathrm{kinetic}\;\mathrm{model}:\;\frac{t}{q_{t}}=\frac{1}{k_{2}q_{e}{}^{2}}+\frac{t}{q_{e}}$$

where *t* is the adsorption time, $q_{t}$ and $q_{e}$ are the adsorption amount at time *t* and the adsorption amount (mg/g) at the time of equilibrium, respectively. *K*_1_ and *K*_2_ are the adsorption constant of pseudo first-order kinetic model and pseudo second-order kinetics, respectively [39].

As shown in Figure 7a, the adsorption rate of MIL-100(Fe) is high in the early stage, and the adsorption equilibrium reached at the time of 20th minute. Similarly, MIL-101(Cr) basically reached at 15th minute as shown in Figure 7b. The fitting parameters of the pseudo first-order kinetics model and the pseudo second-order kinetics model are shown in Table 1. The pseudo second-order model fitted all adsorption process better than that of the pseudo-first order model in terms of correlation factor (R^2^). The fitting degree is high (R^2^ > 0.96) as shown in Figure 7c,d, which proves that the adsorption rate is controlled by the chemisorption mechanism (e.g., hydrogen bond). The fitting results of the pseudo first-order kinetic model is shown in the Figure S5a,b.

At the same time, we fit the adsorption data of different concentrations of aromatic amines on MIL-100(Fe) and MIL-101(Cr). The isothermal models used are the Langmuir isotherm model and the Freundlich isotherm model. The mathematical equations are as follows:

(3) $$\mathrm{Freundlich}\;\mathrm{isotherm}\;\mathrm{model}:\;\ln q_{e}=\frac{1}{n}\ln c_{e}+\ln K_{F}$$

(4) $$\mathrm{Langmuir}\;\mathrm{isotherm}\;\mathrm{model}:\;\frac{c_{e}}{q_{e}}=\frac{1}{q_{m}K_{L}}+\frac{c_{e}}{q_{m}}$$

where $q_{e}$ is the adsorption amount of the adsorbent to the target substance per gram (mg/g), $c_{e}$ is the concentration at equilibrium (mg/L), *K_L_* and *K_F_* represents the adsorption equilibrium constant (L/mg), and $q_{m}$ is the theoretical limit of the adsorption capacity when the surface of the single layer is completely covered [40].

The fitting results (Table 2) of MIL-100(Fe) and MIL-101(Cr) on the adsorption data of aromatic amines with different concentrations are shown in Figure 7e,f and Figure S5c,d. For MIL-100(Fe), although both models showed acceptable fitting goodness for both adsorbents (R^2^ > 0.93), the R^2^ of the Langmuir model was higher than that of the Freundlich model. It seemed that monolayer adsorption could explain this process better. That said, for MIL-101(Cr), the adsorption data of MIL-101(Cr) is more consistent with the Freundlich isotherm model, and the 1/n value is lower (0.5–2), indicating that MIL-101(Cr) is more likely to adsorb 2-nitroaniline and 2-amino-4-nitroaniline, resulting in a larger amount of adsorption [41]. 

### 2.4. Discussion on Adsorption Mechanism

Types and properties of hydrogen bonds acceptor and the appropriate pore systems of MOFs are important factors for the adsorption of specific compounds. De Vos et al. [31] found that MIL-100(Fe, Cr, Al) had good extraction effect on nitrogen-containing compounds. Hou [42] prepared a core-shell structure of Fe_3_O_4_@MIL-100(Fe) to adsorb meloxicam and naproxen. They believed that intermolecular hydrogen bonds between carboxyl in MIL-100(Fe) and secondary amine, hydroxy, and the carboxyl groups are important factors for adsorption. The saturated adsorption capacity of MIL-100(Fe, Cr) and MIL-101(Cr) for five aromatic amines were summarized in Table 3. We noticed that even MIL-100(Fe) and MIL-100(Cr) had same frameworks, their adsorption capacity of aniline, 1-naphthylamine and o-toluidine vary widely. This may be due to the fact that MIL-100(Fe) is more likely to form hydrogen bonds with aniline (1-naphthylamine, o-toluidine). The average bond lengths of the coordination bonds of MIL-100(Fe) and MIL-100(Cr) are ~1.953 Å [24] and ~1.936 Å [43] respectively. And the ions radii of Cr^3+^ (~0.76 Å) is slightly longer than that of Fe^3+^ (~0.69 Å). The average coordination bond length of MIL-100(Cr) is smaller than that of MIL-100(Fe), which indicates that the coordination bonds of MIL-100(Cr) is stronger than that of MIL-100(Fe), and more electrons around the oxygen atom are biased toward Cr^3+^. Therefore, it is more difficult for the coordinated oxygen atoms to form hydrogen bonds with hydrogen from the aromatic amine (aniline, 1-naphthylamine, o-toluidine) than that of MIL-100(Fe).

John R. Morris [44] used UiO-66 to separate the xylene isomers. They found that the diffusion rate of the xylene isomers follows o-xylene < m-xylene < p-xylene. It is proved that the guest molecule and MOF cavity system have an important influence on the diffusion rate. Kolokolov [45] found that benzene can access the pore environment of UiO-66 but the mobility of benzene is severely hindered in the smaller tetrahedral cavities. Wang [29] utilized MIL-100(Fe) to remove diclofenac sodium (DCF) from water. They found that DCF could diffuse into the cavity system through the pentagonal and hexagonal holes of MIL-100 (Fe). For the adsorption of aromatic amines, although each Fe–O coordination bond is a slightly longer than that of Cr–O coordination bond, MIL-100 (Fe) cavity system is more expansion than the MIL-100 (Cr) cavity system, which result in aromatic amines (aniline, 1-naphthaline, o-toluidine) are more easier to diffuse into the cavity system of MIL-100 (Fe) than that of MIL-100 (Cr). Therefore, MIL-100 (Fe) has a larger adsorption capacity for aniline, 1-naphthylamine and o-toluidine than that of MIL-100(Cr).

Yang et al. [32] used MIL-101(Cr) to adsorb volatile organic compounds, including benzene, toluene, ethyl styrene and xylene. Through experiments, they concluded that when the molecular size of the target is appropriate to the pore size of MOFs, MOFs can adsorb more targets. Since nitro group are present in both nitroaniline and 2-amino-4-nitroaniline, the two large oxygen atoms on the nitro group make the nitroaniline (5.1 Å) and 2-amino-4-nitroaniline larger than the aniline. MIL-101(Cr) and MIL-100(Fe,Cr) have their own building units, pentagonal and hexagonal pore windows. The pore sizes of MIL-101(Cr) and MIL- 100 (Fe, Cr) adjacent to their building units are too small (~4.6 Å and ~4.2 Å) for 2-nitroanlinine and 2-amino-4-nitroaniline to enter these pores. These two aromatic amines can only go through the pentagonal and hexagonal pores of MIL-101(Cr) and MIL-100(Fe,Cr). Due to MIL-101(Cr) has larger pore sizes (~12 Å and ~16.5 Å) than that of MIL-100(Fe, Cr) (~5.3 Å and ~8.6 Å), more nitroaniline (or 2-amino-4-Nitroaniline) can enter into the cavity of MIL-101(Cr). For MIL-100(Fe, Cr), part of nitroaniline (or 2-amino-4-nitroaniline) is blocked outside the pores and cannot interact with the hydrogen bond acceptors in the pores, which explains MIL-101(Cr) has the highest adsorption capacity for nitroaniline and 2-amino-4-nitroaniline. This can also explain why MIL-101(Cr) can adsorbs more 1-naphthylamine and o-toluidine than that of MIL-100(Cr).

## 3. Materials and Methods

### 3.1. Materials

All solvents and reactants obtained were analytical grade and used without further purification. Ferric chloride hexahydrate (FeCl_3_·6H_2_O, 99%, Hushi, Shanghai, China), Chromium nitrate nonahydrate (Cr(NO_3_)_3_·9H_2_O, 99%, Hushi, Shanghai, China), 1,3,5-benzenetricarboxylic acid (H_3_BTC, Aladdin Industrial Corporation, Shanghai, China, 98%), Terephthalate acid (TPA, 98%, Aladdin Industrial Corporation, Shanghai, China), Methanol (99.5%, Hushi, Shanghai, China), Absolute ethanol (99.7%, Hushi, Shanghai, China), acetone (99%, Hushi, Shanghai, China) were used to synthesize different MOF samples. Aniline (99.5%, Hushi, Shanghai, China), 2-nitroaniline (98.5%, Hushi, Shanghai, China), o-toluidine (99%, MACKLIN, Shanghai, China), 1-naphthylamine (99%, MACKLIN), 2-amino-4-nitrotoluene (98%, MACKLIN) were used as target aromatic amine contaminants. Milli-Q water (Millipore, 18.25 MΩ∙cm^−1^, Boston, MA, USA) was used in preparation of various solutions throughout the experiment.

### 3.2. Preparation of the MOFs

MIL-100 (Fe) was synthesized with a modified method [46]. FeCl_3_·6H_2_O (2.7 g), 1,3,5-benzenetricarboxylic acid (1.4 g) and 60 mL H_2_O were added to teflon-lined steel autoclaves and maintained at 150 °C for 12 h. After cooling, the light orange solid products were filtered and recovered by two-step method of hot water (80 °C) and ethanol (60 °C) for further purification. After drying at 80 °C, MIL-100 (Fe) was dried at 150 °C overnight in a vacuum [46]. The synthetic details of MIL-100(Cr) [43] and MIL-101(Cr) [47] were shown in Supplementary Materials.

### 3.3. Characterization

A series of analytical techniques were used to characterize the synthesized samples. X-ray powder diffraction (PXRD) patterns were obtained by a X’Pert Pro diffractometer (PANalytical, Almelo, The Netherlands, scanning range 5–50°, Scan step size 0.02°). Thermo gravimetric analysis (TGA, Kejing, Hefei, China) was performed at a heating rate of 5 °C/min in flowing air (40 mL/min) on the SDT Q500 thermogravimetric analyzer. The morphologies and nanocrystalline sizes of MOFs were obtained by scanning electron microscope (SEM) (QUANTA F250, Boston, MA, USA) and transmission electron microscope (TEM) (FEI, Boston, MA, USA). The Brunauer-Emmett-Teller (BET) (3Flex, Atlanta, GA, USA) equation uses a gas adsorption analyzer and measures the total pore volume based on N_2_ adsorption-desorption isotherm. The Fourier transform infrared (FT-IR) spectras were recorded on a Themo Nicolet 6700 (Themo Nicolet, Madison, WI, USA) FT-IR spectrometer using KBr pellets over the range of 4000–400 cm^−1^.

### 3.4. Adsorption Experiments

Aromatic amines were dissolved in ultra-pure water to prepare a reserve solution (100 mg/L). All experiments were conducted in triplicate, the error bars are standard deviations of triple measurements. During the adsorption process, 10.0 mg MOFs was added to 50 mL aromatic amine solution with the required initial concentration (aniline, o-toluidine and 2-nitroaniline (10–60mg/L), 1-naphthylamine (10–70mg/L), 2-amino-4-nitrotoluene (10–80mg/L)). Approximately, 50 mL of solution is dispensed into five glass tubes, shaken on the shaker for 2 h to evenly disperse the MOFs in the solution at room temperature (25 °C), finally mixed. the supernatant was centrifuged. Aromatic amine concentrations were measured at 280 nm using a UV-vis spectrophotometer (Shimadzu UV-2550, Shanghai, China). The capacity of aromatic amine adsorbed by adsorbent per unit mass was evaluated by mass balance equation:

(5) $$Q_{e}=\frac{c_{0}-c_{e}}{m}\times V$$

where *Q_e_* (mg/g) is the capacity adsorbed per gram of adsorbent; *C*_0_ and *C*_e_ are the initial and equilibrium concentrations of aromatic amine in solution (mg/L), respectively; *m* is mass of the absorbent (g) and the *V* (L) is the initial volume of the aromatic amine solution. The calibration curve was obtained from the standard solution.

### 3.5. Adsorption Kinetics Experiment

There was 8.0. mg of MOFs added to a 50 mL (50 mg/L) solution of aromatic amine and shaken at 200 rmp at room temperature. 3.0 mL was taken in batches within 3–90 min, and the supernatant was centrifuged and measured by the above method. Finally, the amount of adsorption was calculated.

## 4. Conclusions

In this work, three MOFs were successfully synthesized and well characterized. The characterization results showed that the three samples have high crystallinity, purity, and applicable surface area and pore volume. They were introduced into an aqueous solution to adsorb aromatic amines. Under the same experimental conditions, the three MOFs showed differences in adsorption for five aromatic amines. Suitable hydrogen bond acceptor, cavity system are two important factors in the adsorption of aromatic amines by MOFs. MIL-100(Fe), MIL-101(Cr) has proven to be a potential metal organic framework material for the removal of aromatic amines from water. Research about the pretreatment of aromatic amine contained samples are ongoing.

## Figures and Tables

**Table 1:** Kinetic parameters of MIL-100(Fe), MIL-101(Cr) adsorption on aromatic amines.

Adsorbents	Aromatic Amine	Pseudo-First-Order Kinetic Model		Pseudo-Second-Order Kinetic Model	
		K_1_	R^2^	K_2_	R^2^
MIL-100(Fe)	aniline	4.29 × 10^−2^	0.783	6.37 × 10^−3^	0.997
	o-toluidine	3.04 × 10^−2^	0.766	8.44 × 10^−3^	0.998
	1-naphthylamine	1.35 × 10^−2^	0.335	5.23 × 10^−3^	0.963
MIL-101(Cr)	2-nitroaniline	5.11 × 10^−2^	0.96	3.86 × 10^−3^	0.997
	2-amino-4-nitrotoluene	9.21 × 10^−2^	0.81	5.96 × 10^−3^	0.997

**Table 2:** Isothermal parameters of MIL-100(Fe), MIL-101(Cr) adsorption on aromatic amines.

Adsorbents	Aromatic Amine	Temperature (°C)	Freundlich Model			Langmuir Model		
			K_F_	n	R^2^	q_m_	K_L_	R^2^
MIL-100(Fe)	aniline	25	5.16	1.58	0.93	84.74	0.0374	0.95
	o-toluidine	25	5.14	1.60	0.94	79.68	0.0369	0.96
	1-naphthylamine	25	7.09	1.85	0.93	72.25	0.0572	0.94
MIL-101(Cr)	2-nitroaniline	25	1.86	1.04	0.93	65.79	0.006	0.76
	2-amino-4-nitrotoluene	25	0.469	1.08	0.97	147.2	0.014	0.43

**Table 3:** Saturated adsorption capacity (mg/g) of aromatic amines by MIL-100(Fe, Cr) and MIL-101 (Cr).

Aromatic amines	MIL-100(Fe)	MIL-100(Cr)	MIL-101(Cr)
aniline	52.0 mg/g	22.7 mg/g	19.6 mg/g
1-naphthylamine	53.4 mg/g	25.8 mg/g	37.3 mg/g
o-toluidine	49.6 mg/g	13.7 mg/g	44.0 mg/g
2-amino-4-nitrotoluene	3.0 mg/g	9.7 mg/g	25.0 mg/g
2-nitroaniline	3.7 mg/g	7.4 mg/g	54.3 mg/g

## Supplementary Materials

The following are available online at https://www.mdpi.com/1420-3049/24/20/3718/s1, Synthesis and activation of MIL-100(Cr) and MIL-101(Cr); Figure S1−S4: FT-IR spectra, TG-DTG curves, PXRD patterns and pore size distributions of the three samples; Figure S5: The calibration curves of (a) aniline (b) 1-naphthylamine (c) o-toluidine (d) 2-nitroaniline (e) 2-amino-4-nitrotoluene; Table S1: surface area, pore volume, pore size of the,MIL-100(Fe,Cr),MIL-101(Cr); Table S2. Thermal analysis data of MIL-100(Fe,Cr), MIL-101(Cr). Table S3: the measurements for each combination of MOF and aniline derivative.
