# Supplementary material for: Metal-Organic Frameworks of MIL-100(Fe, Cr) and MIL-101(Cr) for Aromatic Amines Adsorption from Aqueous Solutions
Source: Molecules. 2019 Oct 16;24(20):3718. doi: 10.3390/molecules24203718 (PMC6832246; doi:10.3390/molecules24203718)
Supplement: Supplementary file 1 [file molecules-24-03718-s001.pdf]

# Supporting Information

# Metal-organic Frameworks of MIL-100(Fe, Cr) and MIL-101(Cr) for Aromatic Amines Adsorption from Aqueous Solutions

Mao-Long Chen*, Shu-Yang Zhou, Zhou Xu, Li Ding and Yun-Hui Cheng*

College of Chemistry and Food Engineering, Changsha University of Science & Technology, Changsha, 410114, China E-mail: mlchen@xmu.edu.cn(ML Chen), chengyh6488@gmail.com(YH Cheng).

Synthesis and activation of MIL-100(Cr) ........ 1
Synthesis and activation of MIL-101(Cr) ........ 1
**Figure S1.** FT-IR spectra of the three samples. ........ 2
**Figure S2.** TG-DTG curves of (a)MIL-100(Cr) (b) MIL-101(Cr) and (c)MIL-100(Fe)........ 4
**Figure S3.** The pore size distribution of MIL-100(Fe) (blue), MIL-100(Cr )(black) and MIL-101(Cr) (red)........ 4
**Figure S4.** The calibration curves of (a)aniline (b)1-naphthylamine (c)o-toluidine (d)2-nitroaniline (e)2-amino-4-nitrotoluene ........ 7
**Figure S5.** (a)(b) The pseudo first-order kinetic model of MIL-100(Fe) and MIL-101(Cr).(c) Isothermal Freundlich model of MIL-100(Fe)(d)Isothermal Langmuir model of MIL-101(Cr). ........ 8
**Table.S1.** Thermal analysis data of MIL-100(Fe,Cr), MIL-101(Cr). ........ 9
**Table S2.** surface area, pore volume, pore size of the,MIL-100(Fe,Cr),MIL-101(Cr). ........ 9
**Table.S3.**the measurements for each combination of MOF and aniline derivative.(mg/g) ........ 10

# Supporting Information

**Synthesis and activation of MIL-100(Cr)**

3.6 g $Cr(NO_3)_3·9H_2O$, 1.35 g 1,3,5-benzenetricarboxylic acid, 60 mL of $H_2O$ were added to teflon-lined steel autoclaves and maintained at 220℃ for 96 h. After cooling, the sample was washed with water and activated by thermal treatment at 150 ℃ [S1].

**Synthesis and activation of MIL-101(Cr)**

Terephthalic acid (1.64 g)，$Cr(NO_3)_3·9H_2O$ (4 g) and $H_2O$ (60 mL ) were added to teflon-lined steel autoclaves and maintained at 220 ℃ for 8 h [S2]. After cooling，the as-synthesized MIL-101 was further purified by hot ethanol, $H_2O$ and acetone solutions. After drying at 80 °C，MIL-101 was vacuum dried at 150 °C overnight.

[S1] Gérard, F.; Christian, S.; Caroline, M.D.; Franck, M.; Suzy, S.; Julien, D.; Irène, M. A hybrid solid with giant pores prepared by a combination of targeted chemistry, simulation, and powder diffraction[J]. Angewandte Chemie Int Ed, 2010, 116: 6456-6461.

[S2] Hwang, Y.K.; Hong, D.Y.; Chang, J.S.; Jhung, S.H.; Seo, Y.K.; Kim, J.; Vimont, A.; Daturi, M.; Serre, C.; Férey, G. Titelbild: Amine Grafting on Coordinatively Unsaturated Metal Centers of MOFs: Consequences for Catalysis and Metal Encapsulation [J]. Angewandte Chemie Int Ed, 2010, 47: 4144-4148.

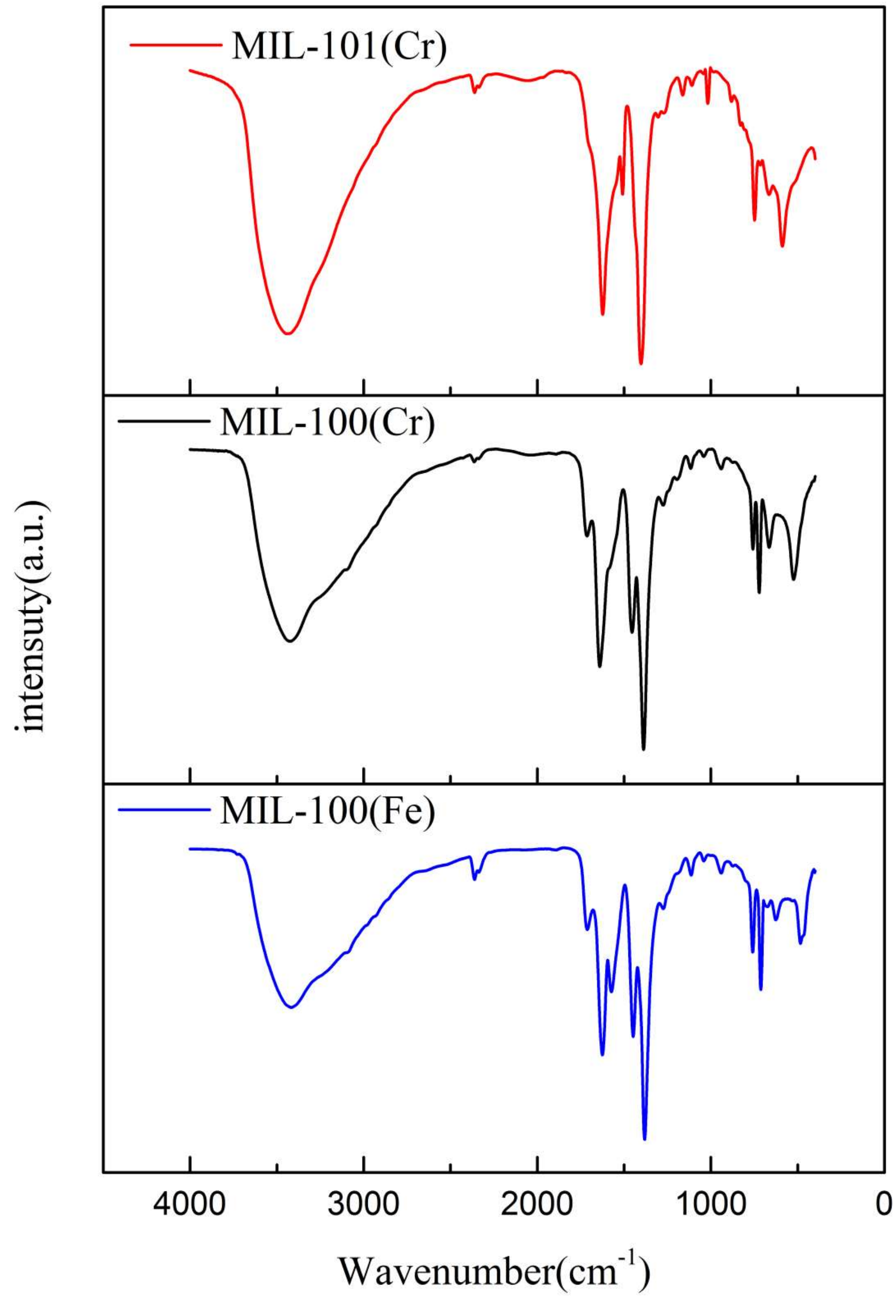


**Figure S1.** FT-IR spectra of the three samples.

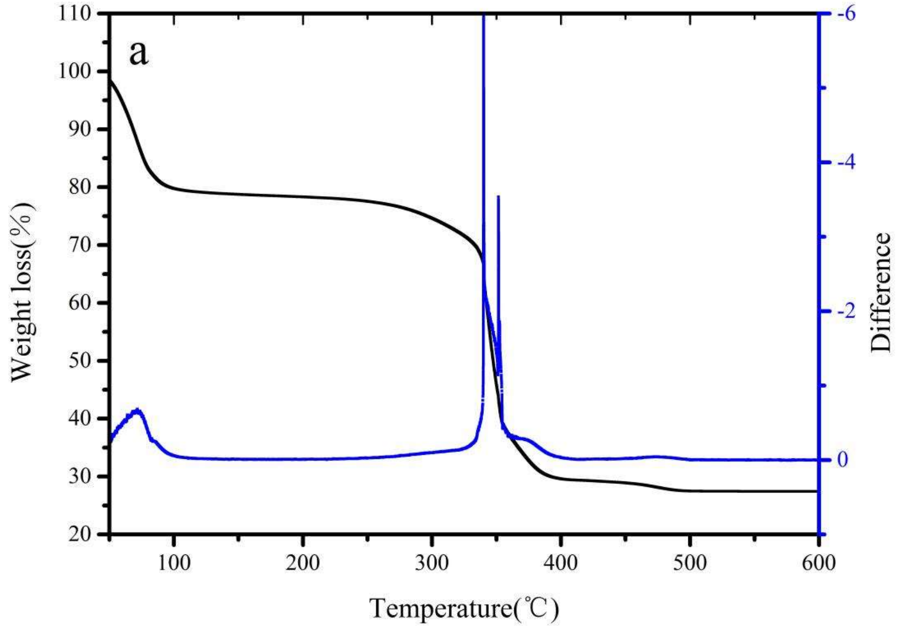
a
Weight loss(%)
Difference
Temperature(℃)

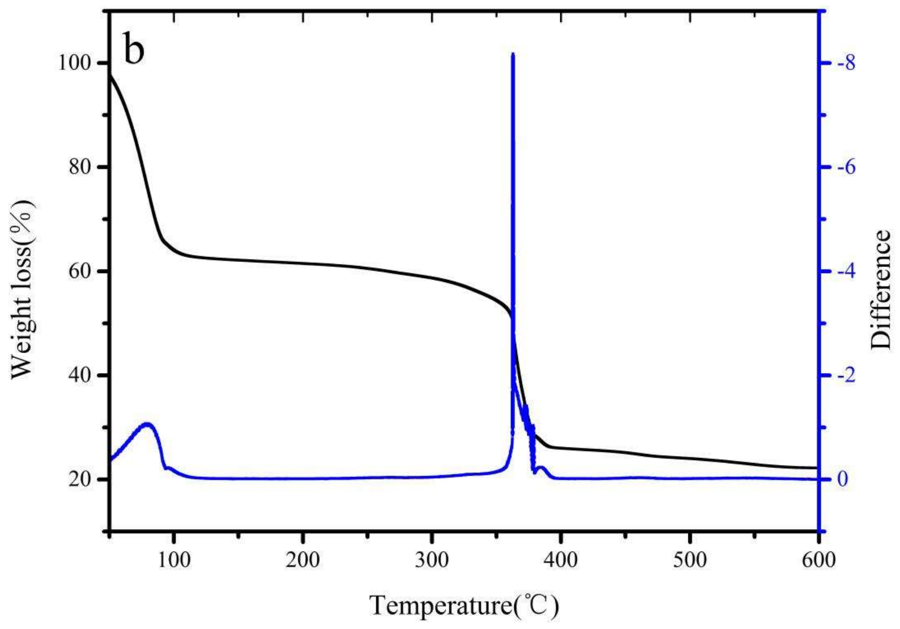
b
Weight loss(%)
Difference
Temperature(℃)

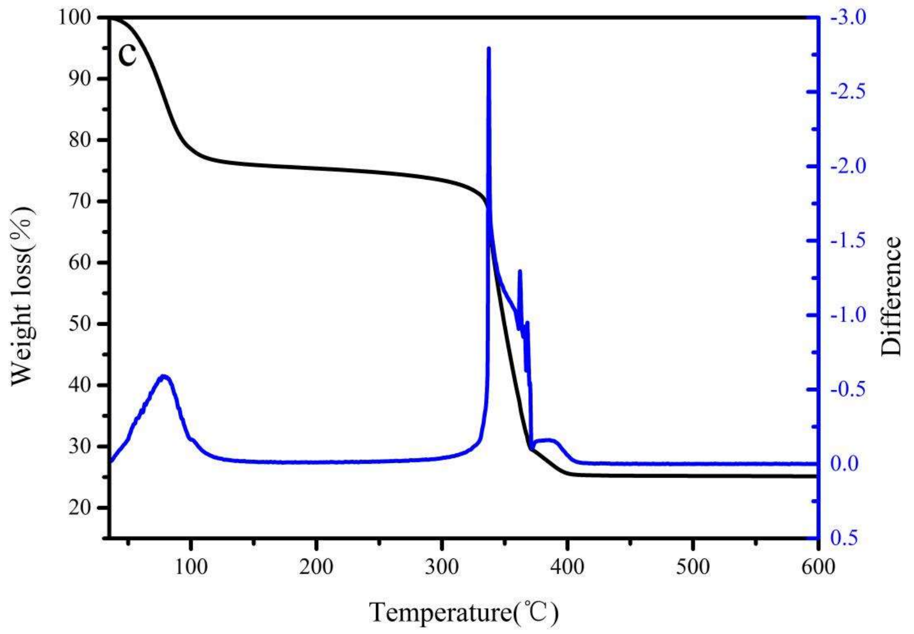


**Figure S2.** TG-DTG curves of (a)MIL-100(Cr) (b) MIL-101(Cr) and (c)MIL-100(Fe)

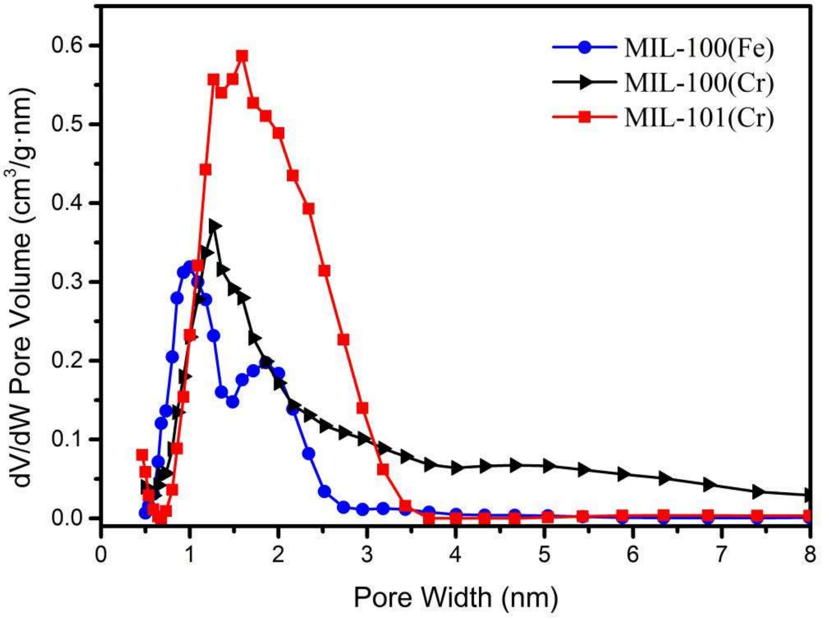


**Figure S3.** The pore size distribution of MIL-100(Fe) (blue), MIL-100(Cr )(black) and MIL-101(Cr) (red).

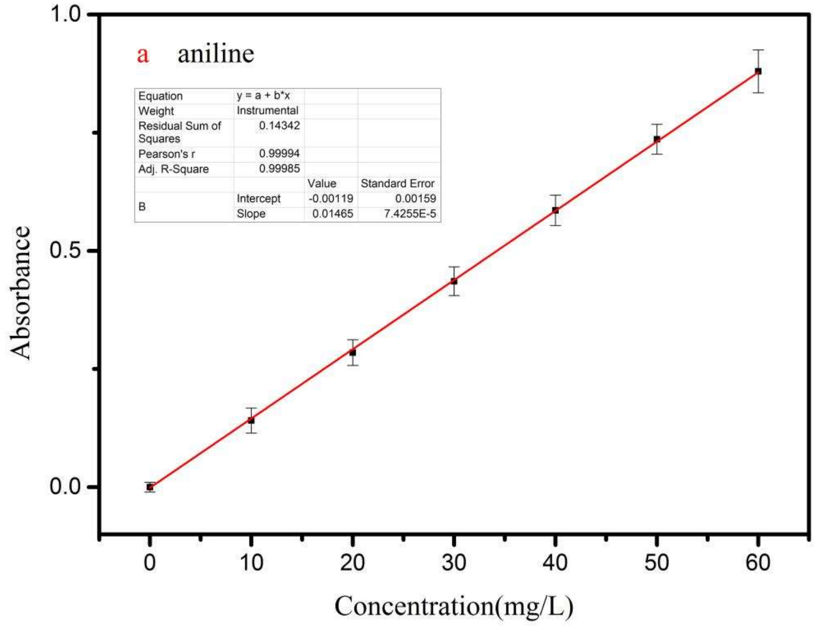
a aniline
Equation y = a + b*x
Weight Instrumental
Residual Sum of Squares 0.14342
Pearson's r 0.99994
Adj. R-Square 0.99985
Value Standard Error
B Intercept -0.00119 0.00159
Slope 0.01465 7.4255E-5
Absorbance
Concentration(mg/L)

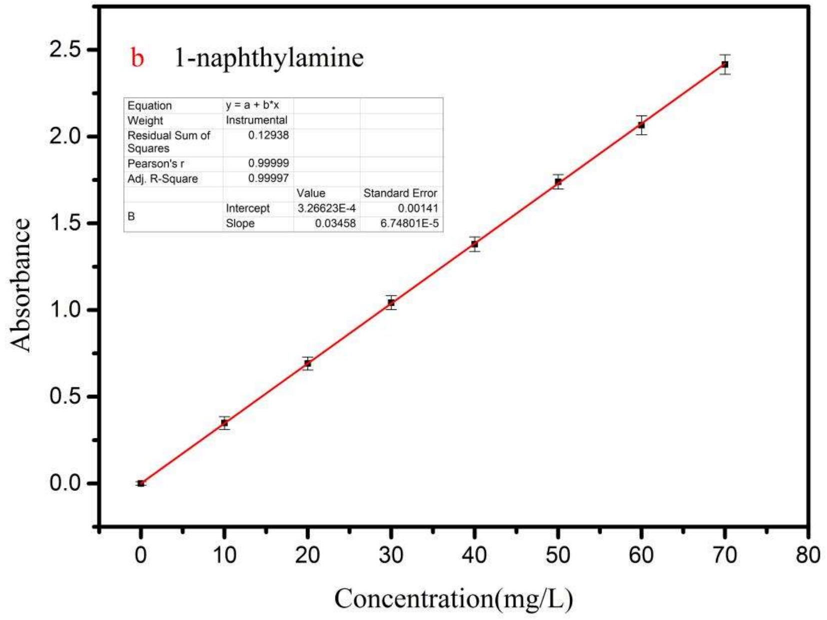
b 1-naphthylamine
Equation y = a + b*x
Weight Instrumental
Residual Sum of Squares 0.12938
Pearson's r 0.99999
Adj. R-Square 0.99997
Value Standard Error
B Intercept 3.26623E-4 0.00141
Slope 0.03458 6.74801E-5
Absorbance
Concentration(mg/L)

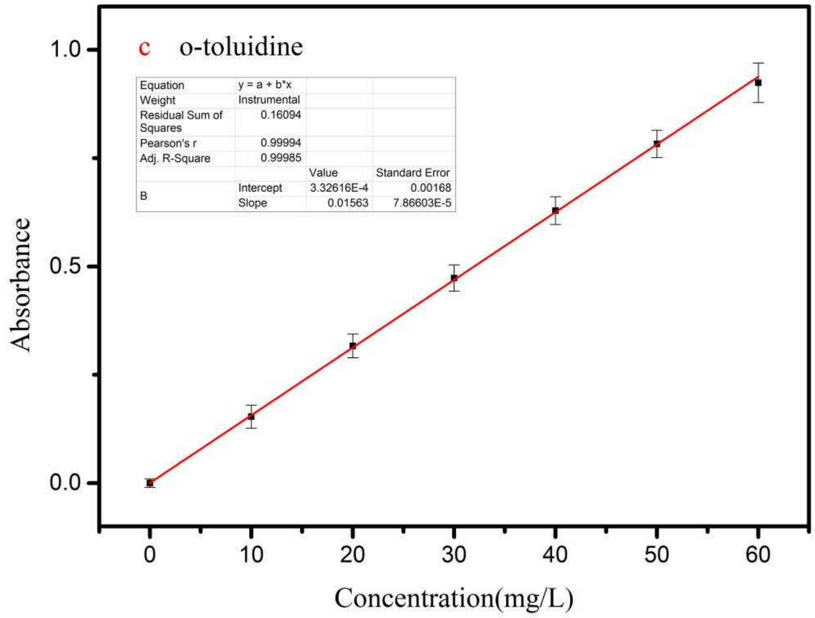
c o-toluidine
Equation | y = a + b*x
Weight | Instrumental
Residual Sum of Squares | 0.16094
Pearson's r | 0.99994
Adj. R-Square | 0.99985
Value | Standard Error
B | Intercept | 3.32616E-4 | 0.00168
Slope | 0.01563 | 7.86603E-5
Absorbance
Concentration(mg/L)

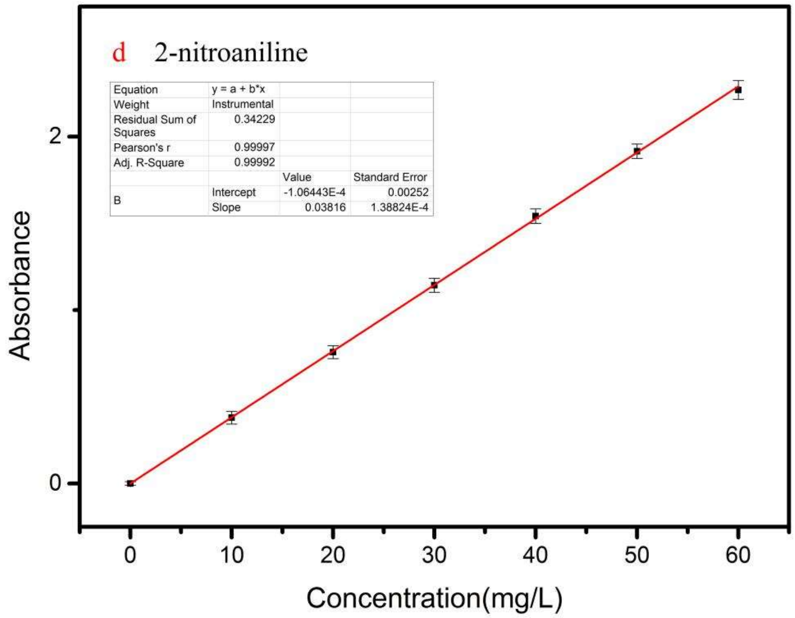
d 2-nitroaniline
Equation | y = a + b*x
Weight | Instrumental
Residual Sum of Squares | 0.34229
Pearson's r | 0.99997
Adj. R-Square | 0.99992
Value | Standard Error
B | Intercept | -1.06443E-4 | 0.00252
Slope | 0.03816 | 1.38824E-4
Absorbance
Concentration(mg/L)

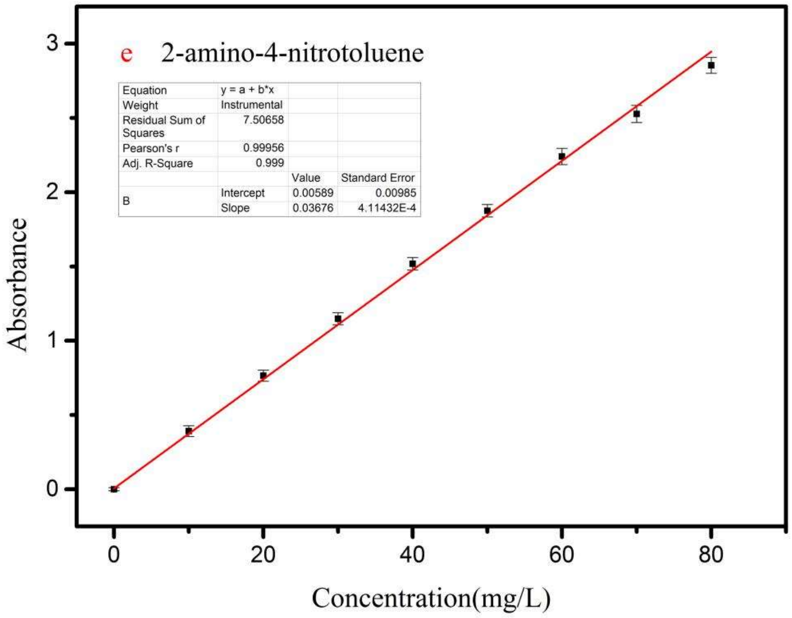


**Figure S4.** The calibration curves of (a)aniline (b)1-naphthylamine (c)o-toluidine (d)2-nitroaniline (e)2-amino-4-nitrotoluene

Aqueous stock solutions of Aromatic amine (100mg/L) were prepared by dissolving Aromatic amine in Milli-Q water. Aqueous solutions with different concentrations(10-80mg/L) of Aromatic amine were prepared by serial dilution of the stock solution with Milli-Q water. The Absorbance of aromatic amine solution were determined using UV-vis spectrophotometer (Shimadzu UV-2550) at 280 nm. Standard curve is established by known concentrations of aromatic amines and absorbance.

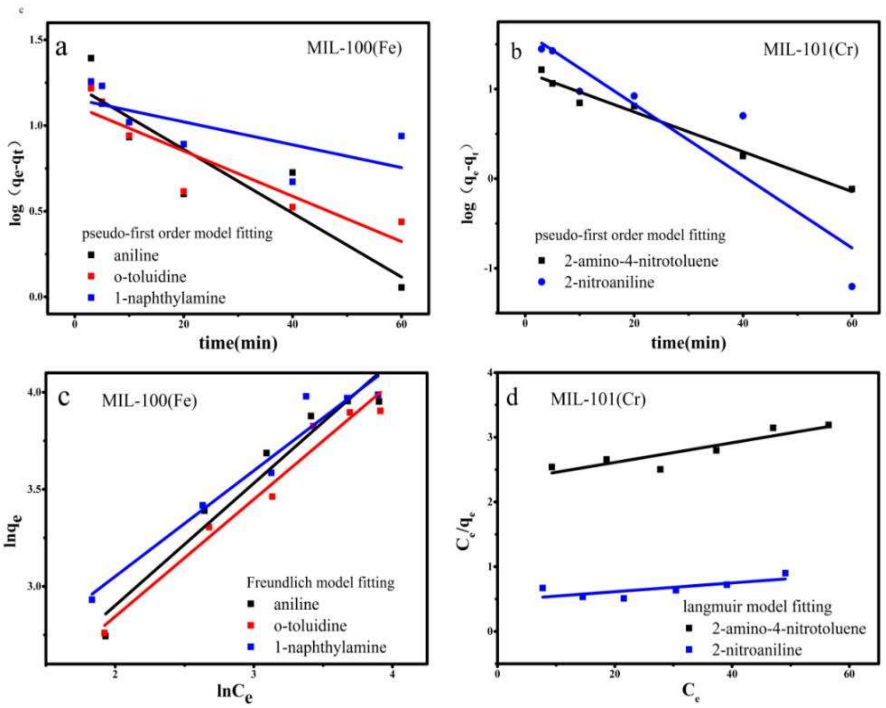


**Figure S5.** (a)(b) The pseudo first-order kinetic model of MIL-100(Fe) and MIL-101(Cr).(c) Isothermal Freundlich model of MIL-100(Fe)(d)Isothermal Langmuir model of MIL-101(Cr).

**Table.S1.** Thermal analysis data of MIL-100(Fe,Cr), MIL-101(Cr).

| Entry | Loss1,water molecules | Loss 2, organic ligands | Calculated residues |
|---|---|---|---|
| MIL-100(Cr) | 76 °C, 79.3% | 341 °C, 27.6% | $Cr_2O_3$, 27.5% |
| MIL-101(Cr) | 81 °C, 62.5% | 362 °C, 22.6% | $Cr_2O_3$, 20.9% |
| MIL-100(Fe) | 84 °C, 75%; | 345 °C, 25.2% | $Fe_2O_3$, 28.5% |

**Table S2**. surface area, pore volume, pore size of the,MIL-100(Fe,Cr),MIL-101(Cr).

| Samples | BET surface area ($m^2$ $g^{-1}$) | Pore volume ($cm^3$ $g^{-1}$) | Pore width (nm) |
|---|---|---|---|
| MIL-100(Fe) | 1018 | 0.67 | 1.14 |
| MIL-100(Cr) | 1427 | 1.39 | 1.26 |
| MIL-101(Cr) | 2134 | 1.15 | 1.61 |

**Table.S3.**the measurements for each combination of MOF and aniline derivative.(mg/g)

| MOFs | Initial concentration(mg/L) | Capacity of aromatic amines by MOFs(mg/g) | | | | | | | | | | | | | | |
|---|---|---|---|---|---|---|---|---|---|---|---|---|---|---|---|---|
| | | aniline | | | 1-naphthylamine | | | o-toluidine | | | 2-nitroaniline | | | 2-amino-4-nitrotoluene | | |
| MIL-100(Fe) | 10 | 13.68 | 15.55 | 17.14 | 18.75 | 19.24 | 17.63 | 15.80 | 14.46 | 17.11 | 0 | 0.21 | 0 | 1.45 | 2.14 | 0.62 |
| | 20 | 27.25 | 29.65 | 31.87 | 30.50 | 31.97 | 28.89 | 27.25 | 28.30 | 30.45 | 0.50 | 0.21 | 0.46 | 1.62 | 0.78 | 2.68 |
| | 30 | 37.27 | 39.90 | 41.07 | 36.05 | 37.26 | 35.08 | 35.25 | 34.15 | 38.35 | 0.50 | 0.43 | 0.14 | 1.76 | 2.77 | 0.98 |
| | 40 | 46.43 | 48.30 | 50.98 | 53.45 | 53.97 | 49.01 | 45.85 | 45.90 | 37.15 | 3.80 | 3.12 | 3.91 | 1.91 | 2.39 | 0.41 |
| | 50 | 49.52 | 52.15 | 54.59 | 52.95 | 56.93 | 48.96 | 49.20 | 50.05 | 42.60 | 3.00 | 3.33 | 2.85 | 2.48 | 1.29 | 2.51 |
| | 60 | 46.06 | 52.05 | 53.97 | 53.90 | 56.75 | 48.71 | 49.60 | 47.40 | 54.45 | 3.70 | 3.31 | 3.60 | 2.67 | 3.37 | 2.08 |
| | 70 | | | | 53.20 | 55.94 | 50.07 | | | | | | | 3.02 | 3.54 | 2.44 |
| | 80 | | | | | | | | | | | | | 2.87 | 3.36 | 2.02 |
| MIL-100(Cr) | 10 | 8.80 | 10.54 | 5.58 | 12.75 | 14.55 | 12.20 | 0 | 0.14 | 0 | 0.52 | 0 | 0.58 | 3.65 | 2.52 | 4.47 |
| | 20 | 13.40 | 14.86 | 9.95 | 20.35 | 19.50 | 16.65 | 2.00 | 2.55 | 1.45 | 1.00 | 1.34 | 0.83 | 4.40 | 5.51 | 3.34 |
| | 30 | 15.40 | 12.17 | 15.83 | 27.25 | 29.30 | 24.50 | 7.80 | 5.55 | 10.15 | 3.35 | 4.21 | 3.22 | 5.35 | 6.53 | 4.19 |
| | 40 | 20.05 | 22.40 | 16.79 | 25.85 | 29.87 | 22.18 | 10.20 | 6.50 | 13.5 | 1.45 | 1.96 | 1.38 | 7.65 | 6.24 | 8.86 |
| | 50 | 22.75 | 25.70 | 18.05 | 25.00 | 32.18 | 20.02 | 13.75 | 8.71 | 17.46 | 7.45 | 5.23 | 7.88 | 7.00 | 5.37 | 8.77 |
| | 60 | 23.80 | 27.93 | 18.76 | 24.50 | 30.36 | 21.08 | 13.05 | 14.5 | 8.15 | 9.30 | 9.68 | 7.61 | 7.30 | 7.79 | 5.37 |
| | 70 | | | | 25.50 | 31.20 | 22.28 | | | | | | | 9.75 | 8.56 | 11.08 |
| | 80 | | | | | | | | | | | | | 7.30 | 7.28 | 9.61 |
| MIL-101(Cr) | 10 | 6.70 | 7.61 | 5.76 | 12.95 | 13.98 | 11.45 | 5.95 | 4.73 | 7.97 | 11.50 | 11.30 | 12.10 | 3.65 | 4.27 | 2.73 |
| | 20 | 14.00 | 15.24 | 10.75 | 25.75 | 26.94 | 24.40 | 21.45 | 21.90 | 14.15 | 27.30 | 28.20 | 26.25 | 7.00 | 6.74 | 7.63 |
| | 30 | 14.15 | 10.07 | 17.91 | 34.50 | 31.50 | 34.50 | 36.4 | 35.15 | 39.30 | 42.25 | 41.80 | 44.85 | 11.1 | 10.31 | 11.75 |
| | 40 | 16.95 | 20.82 | 12.68 | 37.20 | 34.85 | 36.50 | 44.05 | 39.65 | 48.91 | 47.85 | 46.80 | 51.20 | 13.35 | 15.66 | 12.35 |
| | 50 | 19.65 | 21.77 | 17.19 | 37.20 | 42.75 | 33.26 | 44.10 | 42.45 | 49.80 | 54.30 | 54.20 | 59.60 | 14.95 | 13.97 | 15.96 |
| | 60 | 19.30 | 21.88 | 17.37 | 37.35 | 39.74 | 30.90 | 43.60 | 41.10 | 49.30 | 54.55 | 53.15 | 60.25 | 17.7 | 19.23 | 16.37 |

| | | | | | | |
|---|---|---|---|---|---|---|
| 70 | 38.85 | 43.9 | 35.65 | 25.55 | 26.65 | 23.97 |
| 80 | | | | 25.1 | 26.78 | 23.84 |
